# Uncovering Historical Legacies to Contextualize Health Inequities in Puerto Rican Men: An Expansion of the Minority Stress Model

**DOI:** 10.3389/fsoc.2022.830184

**Published:** 2022-02-28

**Authors:** Luis A. Valdez, Anna Mullany, Marielena Barbieri, Aline Gubrium

**Affiliations:** ^1^Health Promotion and Policy, University of Massachusetts, Amherst, MA, United States; ^2^Department of Psychological and Brain Sciences, University of Massachusetts, Amherst, MA, United States

**Keywords:** Latino health, men's health, minority stress, health inequity, thematic analysis

## Abstract

**Background:**

Low/no-income Latino men are disproportionately burdened by chronic disease morbidity and mortality, which is often compounded by persistent exposure to stress. Chronic stress is a key mediating factor in pathways linking macro-level socio-structural forces to micro-level behavioral factors with negative health outcomes. Being that Latinxs continue to be one of the fastest growing populations in the U.S., it is imperative to better understand the roots of stress pathways and explore multi-level interventions.

**Methods:**

This study presents qualitative findings from in-depth interviews with Puerto Rican men (95%) living in Springfield, Massachusetts. We utilized the Minority Stress Model (MSM) first posited by Ilan Meyers, as a framework to understand stress and stress processes amongst Puerto Rican men. We mapped our data onto Meyers' MSM, which allowed us to find diverging themes and identify areas for expansion.

**Results:**

As expected, participants reported stress rooted in experiences of racism and prejudice, expectations of rejection, English-language acquisition, family relationships, insecure housing, precarious employment, and lack of resources. Nevertheless, the MSM did not account for the historical contexts that, as our findings indicate, are used to filter and understand their experiences with everyday stressors. Participants described and linked histories of colonial violence and movement and migration to their stress and community wellbeing.

**Discussion:**

Findings suggest the need to expand the current MSM and our conceptualization of the stress process to include historical understandings when contextualizing present-day stress and future interventions. We propose an expanded heuristic model that delineates the impact of distinctive historical trajectories that aid in interpreting racial health disparities amongst minoritized populations. Future multi-level interventions should give weight to highlighting history and how this impacts the present, in this case including the culpability of U.S. policy regarding Puerto Rico and the adverse health effects for Puerto Rican men on the mainland.

## Introduction

Low/no-income Latino men are disproportionately burdened by chronic disease morbidity and mortality compared to their non-Latinx White counterparts (Freiden et al., 2011; Heron, 2018; National Center for Health Statistics Health United States, 2018). A pressing consideration exacerbating these disparate rates is the emerging data showing Latinxs are at higher risk of hospitalization or death from COVID-19 than other racial and ethnic groups in the U.S. (Centers for Disease Control Prevention, 2020; Sáenz and Garcia, 2021). Higher rates of chronic disease experienced by Latino men are often compounded by persistent exposure to stress (Sternthal et al., 2011; Krieger, 2014). While not all exposure to stress is harmful, a robust body of literature suggests strong associations between chronic stress and poor behavioral, emotional, and physical wellbeing (Stenström et al., 1993; Rosengren et al., 2004; Segerstrom and Miller, 2004; Moreno-Smith et al., 2010). Based on our ongoing research with low/no-income Latino men, we see chronic stress as a key mediating factor in pathways that link macro-level socio-structural forces (i.e., institutional racism, unemployment, and housing insecurity) to micro-level behavioral factors (i.e., detrimental nutrition, sedentarism, substance use, and violence), with negative health outcomes (i.e., diabetes, stroke, liver disease, and psychological distress) (Buchanan et al., 2018; Valdez et al., 2021).

The pathways by which stressful experiences transcend into inequitable health outcomes for Latino men are poorly understood and understudied. Given that Latinxs continue to be one of the fastest growing populations in the U.S., expected to make up 25% of the population by 2050 (U.S. Census Bureau Population Division, 2018), it is imperative to better understand stress pathways and coping mechanisms to inform stress and chronic disease prevention efforts with this community. Utilizing and expanding upon the Minority Stress Model (MSM) developed by Meyer (1995, 2003) as an analytical framework, this article uses qualitative data from 40 in-depth interviews with Puerto Rican men to deepen our ability to understand and intervene upon the etiological pathways of stress and stress processing that is uniquely experienced by this subset of Latino men.

While understandings differ across disciplines, for the purposes of our work, we operationalize *stress* as the “*the process in which environmental demands tax or exceed the adaptive capacity of an organism, resulting in psychological and biological changes that may place persons at risk for disease”* (Cohen et al., 1995). That is, stress-inducing exposures can include routine environmental pressures related to family or work, significant life changes, traumatic events, social isolation, exposure to violence or natural disasters, as well as lived and vicarious experiences with racism and discrimination (Valdez et al., 2021). A recent review suggests that experiences with discrimination are associated with alterations in hypothalamic-pituitary-adrenal (HPA) axis activity; timing and duration of discrimination experiences may be central to understanding how this leads to HPA dysregulation resulting in stress-related disease (Agorastos and Chrousos, 2021). Persistent psychosocial stress related to discrimination also has been associated with elevated systolic blood pressure, increased body fat, and higher fasting blood glucose levels (Williams et al., 2003; De Vogli et al., 2007), effects that may differ by gender for Latinxs (McClure et al., 2010). Fear of persecution due to immigration authorization status, exposure to unjust policing practices in minority communities, being denied employment or housing, and receiving inadequate education or medical services can lead to stress for Latinxs (Finch et al., 2000; Berk and Schur, 2001; Pérez et al., 2008; Araújo Dawson and Panchanadeswaran, 2010). Inclusively, social isolation-related psychological distress often compounds stressful situations that Latinxs find themselves in, particularly when considering migrant Latinx populations (Negi, 2013). Social isolation among migrant Latino men has been associated with higher depressive symptoms, as well as drug-involved accidental death (Mora et al., 2014).

Research shows Puerto Ricans, generally speaking, experience higher rates of psychiatric disorders and poor physical health in comparison to other subgroups of Latinxs (Alegría et al., 2007; Wassertheil-Smoller et al., 2014; Woo et al., 2020). For instance, findings from the Hispanic Community Health Study (*N* = 15,830) suggest that Puerto Rican men, specifically, had the highest mean allostatic load in the sample, which increased with age (Salazar et al., 2016). Accordingly, findings from the National Latino and Asian American Study suggest that Puerto Rican Latino men experience the highest rates in 8 of 15 stress-related physical ailments, including heart disease, hypertension, and overweight and obesity, compared to their Non-Latinx White counterparts and other subgroups of Latino/a men and women (Ai et al., 2013).

Research also indicates that movement and migration, whether from island to mainland or state to state within the mainland, is associated with increased psychological distress and poor physical health for Puerto Rican Latinxs (Diaz, 1998; Aranda, 2007; Gonzalez et al., 2021). “Colonial migration”, as McGreevey (2018) names it, has been and continues to be an integral part of Puerto Rican life. Colonial migration is undertaken for survival: U.S. colonial policies in Puerto Rico result in political, social, and economic upheaval and drive Puerto Ricans to search for higher wages, education, and access to social services (Vasey and Manderson, 2010; Lueck and Wilson, 2011; Gonzalez et al., 2021). Major shifts in migration toward the continental U.S. and the formation of the Puerto Rican diaspora began shortly after World War II, as U.S. industrialization spread into Puerto Rico under “La Operación Manos a la Obra”, (Operation Bootstrap), beginning in 1947 (Santana, 1998). The policy included tax exemptions for U.S. corporations to set up factories on the island, with the provision of a cheap labor force and under the premise of “building a modern, developed nation” (Santana, 1998, p. 93). Imperialist policies shifted the economy from agriculturally-based to primarily export manufacturing. This shift led to rural dislocation, family disruption, quelling of independence movements, increased economic dependency on the U.S. and forced reliance on imports, and the drive of migration North in hopes of a better life (Silén, 1971; Santana, 1998; González, 2011).

Although this migration as a result of Operation Bootstrap was known as the “Great Puerto Rican Migration” (1950–1960), the years from 2006 to 2017 saw the largest Puerto Rican migration to the mainland in history, with ~54,000 Puerto Ricans migrating annually (Gonzalez et al., 2021). Economic actions by the U.S. (supported by Puerto Rican officials) continued to devastate Puerto Rico, leading to an increase in the island's public sector debt and enhanced austerity measures. Poverty, unemployment, and food insecurity rates all saw major increases, along with crumbling infrastructure, increases in crime, and hollowing out of any social safety nets (Gonzalez et al., 2021). More recently, Hurricane Maria and a series of earthquakes have devastated the island, driving more current migration from the island to the mainland. Furthermore, movement within the U.S. also stems from disinvestment within communities and higher costs of living, leading Puerto Ricans on the mainland in search of better opportunities in other states (Krause and Gubrium, 2019). Currently, the ongoing pandemic has deepened the already crisis-laden economy and it is predicted that 300,000 Puerto Ricans will migrate to the U.S. mainland between 2020 and 2022 (Segarra, 2020). The center holding piece of Puerto Rican migration was and continues to be rooted in U.S. colonialism—the proliferation of destabilizing pre-migration conditions in Puerto Rico is intimately linked to the accumulation of U.S. profits and geo-political power. On the individual level, the ongoing colonial status of Puerto Rico has and continues to have a disastrous and chronic effect on the mental health of Puerto Ricans (González, 2011).

In an effort to better understand the etiological pathways of stress and stress processing uniquely experienced by Puerto Rican men within our study, we reference the MSM originally posited by Meyer (1995, 2003). The MSM is a heuristic framework that elucidates the potential pathways that lead from chronic stress exposures and health outcomes in vulnerable groups. The MSM, developed by Meyers, is frequently used to explain heightened psychological distress and greater risks of suicidality in gay, lesbian, and bisexual populations (for example: Flenar et al., 2017; Kramer et al., 2017; Avery-Desmarais et al., 2020). The core concept of the MSM establishes why stigmatized and marginalized populations experience disproportionately higher rates of chronic stress and related adverse health effects due to their social “minority” status. Stress stems largely from internalized marginalization, perceived stigma, and actual events of discrimination and violence (Meyer, 1995). The model includes both proximal and distal influences to illuminate the processes of stress within a marginalized population, while helping to visualize both the myriad stress-related circumstances in a particular population's experience and protective factors that reduce stress. Further, the MSM includes such variables as environmental circumstances, mental health outcomes, general life stressors such as marital disputes or finances, identity characteristics specific to marginalized groups, and coping and social support mechanisms to combat stress.

Although often used for health intervention measures aimed at the LGBTQ community, the MSM has since been utilized to examine and explain chronic stress and adverse health in other marginalized populations, as originally envisioned by Meyers. For example, the MSM model has been used in studies examining the experience of autistic individuals struggling with mental health (Botha and Frost, 2020), stress and isolation in Latino day laborers (Negi, 2013), and stress and food-related practices within immigrant populations (Berge et al., 2018). Applying the MSM to historically marginalized social groups (as in these examples) is useful for locating the multi-level manifestations of stress for a particular “minority identity”, providing descriptions of environmental factors and institutional discrimination, as well as discovering protective factors.

Meyers argues for the need for multi-level interventions. “The stress model”, (Meyer, 2003) contends, “can point to both distal and proximal causes of distress and to directing relevant interventions at both the individual and structural levels” (p. 692). Overall, the model posits that stress can be conceptualized as subjective (individual) and objective (structural) (Meyer, 2003). The subjective view considers how an individual copes with stress and personalized experiences, such as internalized stigmatization or being the recipient of a discriminatory act; the objective view pays attention to the stress-inducing environmental factors, like pollution or crumbling infrastructure, that force an individual to adapt and are present regardless of individual reaction. (Meyer, 2003) considers the assumptions that have long been made about minority stress by researchers, namely, that minority stress is: (1) unique (added stress based on marginalization); (2) chronic (reoccurring); and (3) socially based. Socially based relate to the “objective view” that stress is created due to “social processes, institutions, and structures” (i.e., discrimination within courts, educational systems, and housing policies). Here, however, is where Meyers' model falls short, particularly when considering structural or “objective” circumstances. Absent is recognition of the historically-based circumstances of one's life that could help explain discrimination-based stress and maintenance of the status quo, for any minoritized population. Krieger (2014) writes that discrimination at its core is “a historically entrenched cross-generational societal phenomenon, one that creates and preserves privilege for dominant groups at the expense of subordinated groups. After all, if discrimination served no function, it would presumably be simple to eliminate” (p. 687). The MSM—as it is—can illuminate the privileges that dominant groups possess, because the model enables the parsing out of society's institutions and their impact on marginalized identities. However, it overlooks the “historically entrenched” component that Krieger gives a nod to.

This article discusses how the MSM can be expanded to include historical legacies and then utilized to examine stress-related health disparities of minoritized populations. We propose an expanded heuristic model that delineates the impact of distinctive historical trajectories that aid in interpreting racial health disparities amongst minoritized populations. Importantly, the objective of this work is not to critique the shortcomings of the MSM but to expand upon its carefully constructed architecture to add dimensions that could amplify its utility with other minoritized populations. The intended use of our model is not only to better understand the etiology of disparities in disease outcomes, but also to provide a pathway toward developing improved responsive multilevel intervention efforts with Puerto Rican men.

While the terms Latino and Hispanic are often used interchangeably to describe people of *any race* with cultural ties to Latin America, we have chosen to use Latino (and the variant Latinx) as it is the most widely recognized within the communities where the current work took place. The term Latinx (plural Latinxs) is a neologism used to describe people of Latin American ancestry in a gender-inclusive manner (María del Río-González, 2021). For the purposes of our work, when referring to a community as a whole where the gender of a collective is unknown, we use *Latinx*. When referring to subgroups in which self-reported gender is present we use *Latino* or *Latina*.

## Methods

The Men of Color Health Awareness (MOCHA) program is a community driven effort started in 2012 in Springfield, MA. While MOCHA's approach has evolved throughout the years since its inception, in its latest rendition, MOCHA has brought together cohorts of low/no-income, mostly African American men, with an aim to improve health through participation in a 10-week program to decrease stress and chronic disease vulnerability. MOCHA does this through an integrated model that addresses physical, mental, and spiritual health, while also emphasizing social connectedness and understanding of stress rooted in experiences with poverty and class discrimination and racism, and gender role strain (Valdez et al., 2021). The data used in this study were collected as part of a formative effort to improve the cultural responsiveness of the MOCHA curriculum for Latino men in the greater Springfield, MA area.

### Study Setting

All data collection occurred in non-clinical community locations in the greater Springfield, MA metropolitan area between October 2019 and January 2020. This area struggles with the effects of deindustrialization and a precarious economy, which has a disproportionate effect on the socioeconomic survival of its most vulnerable citizens (Mullany et al., 2021). To date, Latinxs comprise ~49% of the Springfield, MA metro area (City Data, 2019), which is the largest population of Puerto Ricans per capita on the mainland U.S (Granberry and Mattos, 2019). Latinxs comprise 28% of those living below the poverty level, over double that of the non-Latinx White population (City Data, 2019). Latinxs in the area experience the compounded effects of economic and educational disinvestment, high unemployment rates, persistent racial segregation, lack of access to adequate housing and transportation, environmental exposures, food insecurity, and lack of political representation. As a result, Latinxs living in the Springfield metro area are disproportionately burdened with health inequities (Cortés and Vega, 2010).

### Recruitment and Participants

Active recruitment took place *via* project-based tabling at community agencies, local employers, and other relevant community events coordinated by a Latinx bi-cultural, bi-lingual, male-identified MOCHA mentor (i.e., graduate of the MOCHA program). Men were eligible to participate if they: (1) self-identified as a Latino/x, Hispanic, or bi-ethnoracial-including-Latino man; (2) aged between 25–64 years; (3) self-defined insufficient or low/no-income; and (4) lived in the Springfield area for the previous 6 months. A total of 48 men were scheduled for interviews, and 40 of those men completed interviews and sociodemographic questionnaires.

All but three of the participants were of Puerto Rican origin. The average age of participating men was 57.5 years. Approximately 30% of the men were married or lived with a spouse, 55% did not obtain a high school diploma (or equivalent), and 90% men reported < $29,999 in annual income. Over half (55%) of the participants spoke only Spanish, and 72.5% were born in Puerto Rico. All study procedures were approved by the University of Massachusetts Amherst Institutional Review Board.

### Data Collection and Analysis

A research team member (LAV) trained in community-engaged qualitative data collection carefully explained all study procedures to participants and provided space and time for questions and needed clarifications. The semi-structured, in-depth interview protocol was based on questions used in inquiry conducted by the research team in formative work for previous renditions of the MOCHA curriculum and elicited the examination of: (1) definitions of health, (2) manhood/masculinity and being Latino/x, (3) perceptions of stressors and coping mechanisms for Latinxs, and (4) perceptions of the fairness or justice of health disparities. Nevertheless, the majority of relevant data used in this analysis emerged from discussions of *stressors and coping*. The interview protocol included questions such as “What does ‘being healthy’ mean to you,” “Tell me a little bit about what you learned, while growing up, about how to be a man,” and “Thinking back over the last week or two, what sorts of stresses (if any) did you experience?” Participants were carefully informed of all study protocols, as well as the risks and benefits of their participation, and were given ample time and space to ask questions before their written and verbal consent were obtained. Recorded interviews lasted anywhere from 60 to 90 min and were followed by a sociodemographic questionnaire. Participants received $25 for their participation in the study.

Digital recordings of each interview were transcribed verbatim. Interviews that were conducted in Spanish were translated into English to facilitate analysis, which included a back translation process to ensure the validity of the translation and to limit potential loss of meaning. Because the primary purpose of the current study was to better understand stress and the stress process of Latino men, we used the Minority Stress Model (Meyer, 2003), first as a heuristic device for mapping our data, and then as a guide for our coding process. We used a hybrid thematic analysis approach (Fereday and Muir-Cochrane, 2006) to facilitate the use of a priori themes based on the objectives of our work, as well as the identification of themes that emerged throughout our analysis. Two (LAV, AM) members of the research team trained in qualitative data analysis reviewed the transcripts for accuracy and developed an initial codebook. Thereafter, three (AM, SS, MB) members of the research team conducted iterative reading and preliminary coding of four transcripts and came together to settle any coding disagreements. Coded transcripts also were spot-checked by one member of the research team (LAV) to ensure reliability of coding and to account for analytic drift. Theme saturation was derived from the diminished variation in coded transcripts. NVivo 13 (QSR International) was used to facilitate data organization and management.

## Results

**Figure 1:**
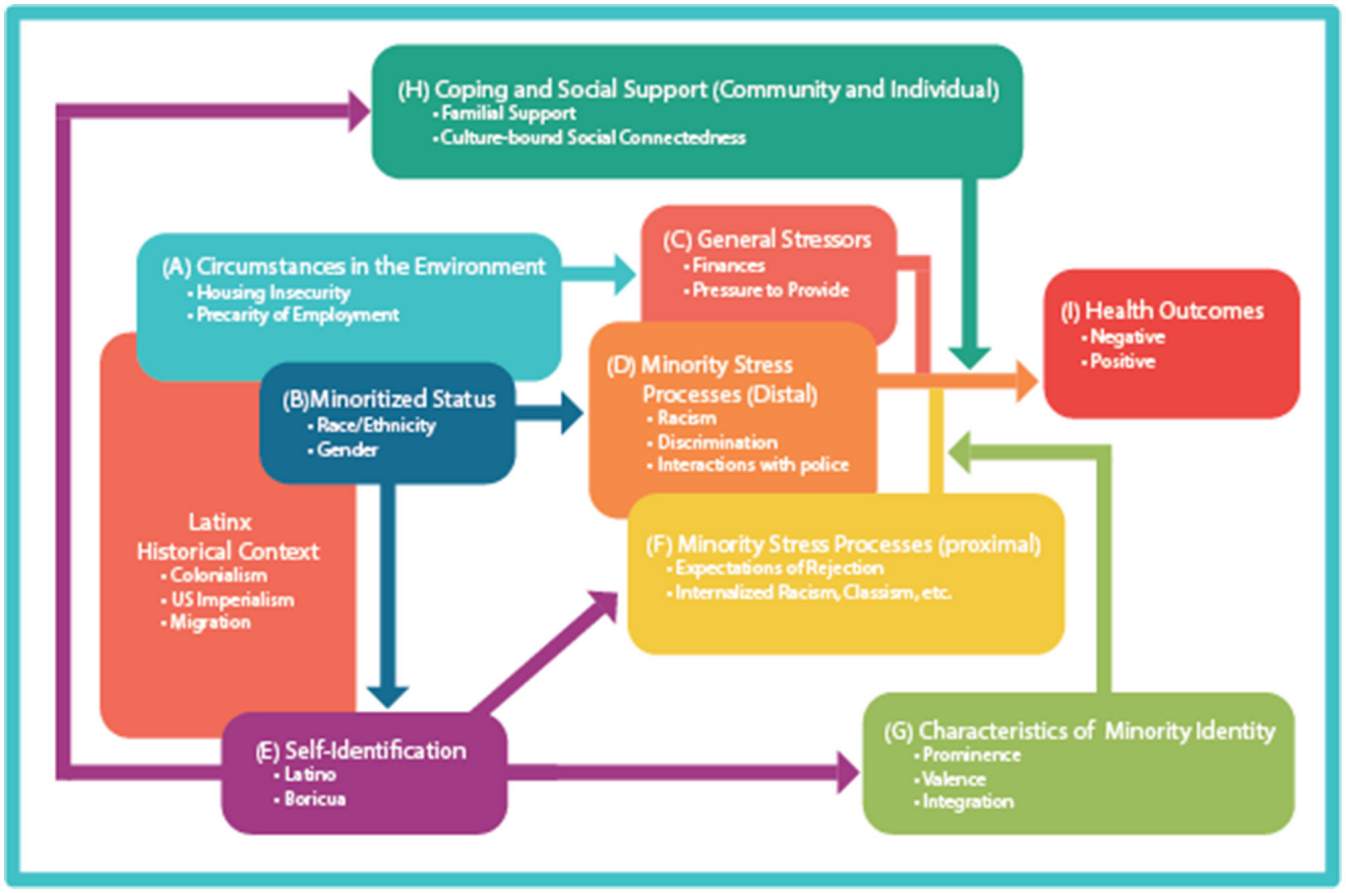
Expanded minority stress model adapted for Latino men.

The MSM assisted in identifying both structural correlates and more individualized psychological stress processes and patterns, adverse mental and physical health outcomes, and coping mechanisms; all these variables were understood and linked to the men's “minority status” of being Puerto Rican. We found, however, that in mapping our data, there was a gap in the MSM that did not historicize the men's lives and make visible the life histories and trajectories that contribute to and contextualize current stressors. Most notably, interspersed throughout the interviews the men reflected on both the colonial status of Puerto Rico and their lived experiences with movement and migration. In the first part of our results, we illustrate our utilization of the MSM (Figure 1, 2003) in parsing out the data to show the stress processes of marginality and coping. In the second part of our results, we use brief excerpts from our interviews to illustrate the need to expand the model to include consideration of historical contexts of stress for Latino men. Figure 1 is an adaptation of the Minority Stress Model based on that originally posited by Meyer (2003) and includes how our data from Latino men map onto the original elements of the MSM along with our addition of the historical context.

### Mapping Onto the MSM

#### Surviving at the Margins

In congruence with existing minority stress models (Meyer, 2003), stressors and the stress process reported by the men in our sample were *uniquely* experienced by them (not experienced by non-stigmatized populations), *chronic* (repetitive over a long time), and *socially based* (amplified by social process, institutions, and structures). As expected, iterative analyses of this data suggested that, to a large extent, the men in our sample reported stress rooted in experiences of racism and prejudice, expectations of rejection, English-language acquisition, family relationships, insecure housing, precarious employment, and lack of resources. Direct mapping onto the MSM facilitated a clear visualizing of the stress processes for these men. For instance, in terms of (*b/e) the minority status and identity*, the participants were keenly aware that the stressors they experienced were due to the intersection of their identities as Puerto Rican, as men, their marginalized socioeconomic status, and for some, previous experiences with the criminal justice system, all of which resulted in exacerbated social marginalization. The men in our sample reported that some of their most concerning stressors were related to direct and vicarious experiences with prejudice and discrimination due to their physical features (e.g., skin tone and hair), their nativity, and their English language acquisition. Importantly, men in our sample, largely U.S. citizens, reported having been victims of direct discrimination due to the compounded effect of being believed to be undocumented at a moment in time when the political discourse was fervently scapegoating and vilifying undocumented Latinx im/migrants.

Many of the men discussed *(a) environmental factors* that induce stress, such as gang activity in their neighborhood, lack of community resources, precarious employment, and/or substandard housing. Men also were keenly aware of their *(b) minoritized statu*s and the MSM helped to plot out the men's intersected *(c) general* life stressors, such as troubled finances, dealing with death, and marital strain. For example, participants recalled that many of their stressors are linked to their ability to provide for themselves and their families. Men also reported that this stress often is compounded due to their perceived lack of sustainable employment opportunities, citing that most of the work available to them pays little, is often strenuous and dangerous labor, or is temporary or precarious. Men discussed *(d) distal*-induced stress that stems from racialized interactions embedded within institutions, such as policing, a disinvested education system that yields poor educational opportunities and outcomes, and housing and employment discrimination. And finally, analysis of our data based on the MSM illustrated clearly how direct racist stigmatization toward Latinx im/migrants (distinctly Black and Brown migrants) plays out in both internalized and external ways; these (*f*) *proximal stressors* included chronic individual experiences with race- and ethnicity-based discrimination, intra-group disunity, self-stigmatization, and internalized expectations of failure.

#### Social Connectedness and Ameliorative Coping

Importantly, the MSM also highlights coping mechanisms and supports bound to participants' minority status as Latino men that buffer stress. The creation and nurturing of social connectedness, centered in familial ties and otherwise, was highlighted as an imperative factor in *(h) coping and social support* for participants. Specifically, the men in our sample reported how culturally significant family relationships, positive interpersonal relationships with others, and overall community support were integral to buffering the impact of daily and chronic stress. For instance, characteristics of family relationships, such as feeling responsible for their family and overall perceptions of connectedness with family members, allowed them to cope and promoted increases in positive psychological wellbeing. The men also coped with stressors by helping others in need, as well as promoting friendly personal engagements with others as a way for them to increase their wellbeing and overall sense of self-worth. Being respectful of others, especially their elders, and expressing that respect through lending a helping hand, were important values and behavioral acts for the men. Being selfless not only influenced their ability to cope with their daily lives, but their perceived benevolence aligned well with their religiosity, which also improved their psychological wellbeing and capacity for coping.

Participants also discussed how support within the community is a protective mechanism against daily life stressors, and subsequent negative psychological and societal outcomes. The men emphasized the importance of providing informational support to others by supplying information on resources and programs within the community that can supply fundamental support when needed. For example, the men promote programs like MOCHA in hopes that another person may benefit from obtaining the support and resources (i.e., employment, food, housing, and therapy) supplied by these community organizations.

Lending support was not only important to their sense of self at an individual level, but also important to their perception of community in general that was bound to their identities as Latinxs. The men spoke about the pride they felt toward their own culture and people, as well as pride in their collective ability to come together to provide support to others who may be in need. The men expressed the importance of providing aid to other Latinxs within their community to overcome the societal barriers they may encounter (i.e., language barriers, lack of transportation). Overall, the men agreed that providing support at individual and community levels, as well as their overall social connectedness through specific relationships, influenced positive coping behaviors in the face of stressors.

### Expanding Minority Stress Models: Inclusion of Historical Contexts

Our findings highlight important deviations from the stress process considerations posited by the MSM. Particularly, interview-triggered discourse about stress, racism, and discrimination also elicited discussion about men's experiences with movement and migration, especially as it related to Puerto Rico's deleterious history of colonization, continued territorial possession of the island, the men's perception of their standing as second-class citizens in the U.S, and paternalism. As one participant put it: “We're the adoptive children of the United States.” When asked to discuss the stressors that may be unique to Latino men living in the U.S., participants at times filtered and contextualized day-to-day stressors through an understanding of the U.S.-Puerto Rican political dynamics that have resulted in the diasporic and often substandard conditions of Puerto Ricans living on the mainland.

Several men highlighted the larger violence of colonization and economic impact of ongoing U.S. policies. One man considered racialization and violence in light of imperial subjugation: “So why are there White Mexicans? Why are there Brown Mexicans? Why is the Boricua Brown? Why are there Boricuas Latinos? Because of the violence.” This violence he refers to is the history of conquest by imperial powers—first from Spain, then the U.S. Another participant reflected on violent conquest as he spoke about his pride for Puerto Rican culture:

But then there is a downfall to [Puerto Rican pride] also because [our history] doesn't make you feel proud. Because Christopher Columbus killed Puerto Ricans. He made slaves out of Puerto Ricans. He stole and raped Puerto Ricans. And they celebrate his birthday, they have statues of him. And he was a cold-blooded thief and a murderer.

This abhorrence of Columbus' legacy was echoed by another participant and linked to other Native plunder: “Do you know what the crazy part is? White people were not the first ones here, Native Americans were...Yeah, but they…They killed… They took America away from them.”

Participants discussed different forms of impoverishment on the island that they saw as linked to continued economic pillage and possession of the island. One participant referenced the current debt crisis: “you know we owe like thirteen or fifteen trillion dollars to the United States from Puerto Rico.” In the next sentence he remarked on the large number of Puerto Ricans who have migrated from the island to the mainland: “Why don't they just make it another state? Everybody is over here. Holyoke is Puerto Rico, Springfield is half Puerto Rico, and Westfield is half Puerto Rico.” Another participant linked the crumbling infrastructure in Puerto Rico left by years of U.S. disinvestment in the post-industrial era to its now substandard medical system and consequent impact on health:

Why do the Latino have a higher rate of chronic diseases? Let's say, more than the White men in the United States.... See, to me it has to do from where we came from, because we come from an island...The United States has better hospitals, better medicine.

Another participant further reflected on the U.S. acquisition of Puerto Rican resources while failing to invest in its people: “How are you going to come to my land to establish business, to steal from us...and you going to slap us in the face also?”

Along with historical legacy, participants punctuated their interviews with stories of often difficult and violent life experiences directly or indirectly linked to migration from the island to the mainland and movement within the U.S. The stories foretold how these experiences have shaped their current realities:

I came from Puerto Rico when I was fourteen. To better my family. My father was never with us. I had to help my family when I was in Puerto Rico. I left school to help my family, I worked for them. Until I came here, then slowly I started sending for them one by one… I started working, I had my own apartment. I sent for my mom, my little brothers, the little ones that were left...thank God, until I brought them all. We started a new life out here…

Similar to the story above, another participant also moved to the mainland while young and took care of his family:

I came here when I was 10 years old. To Brooklyn, a real bad place… Life for me…was a draw. Yeah because from where they took us, they took us to a jungle you know. Something horrific … We moved to Pennsylvania and the things that happened to my mother and the rebellion that I saw in the world. I was rebellious to the world, because of the things that happened to us, that happened to my mother.

One man spoke of the suffering he experienced here in the U.S.—although the “Island is bad,” it is “a thousand times [better] there.” He stressed—“You suffer. You suffer. You suffer here.”

One repeated type of suffering centered on language acquisition and treatment as second-class citizens. One participant reflected: “A lot of the White people are racists. When they hear the accent, you notice it. The majority, the first thing they say is ‘We are in America, not[exp] Puerto Rico.” Another participant spoke of expectations on the mainland that exacerbate stress:

The White man is from this country. Not us. We have to get accustomed to this country. We have to learn their language...and we have to learn their rules … And we were not born with those rules, we learn them here. It's harder. It's not your place. You can't do whatever you want like in your country. There are different rules, different norms, different politics. Everything is different.

Lastly, another participant's response exemplified how the men sensed their own second-class status on the mainland:

It damages your mind… In the United States it is difficult to be Latino, because, because there's, there's… the matter of humiliation. But if we think about it, we are part of the United States. Because, right away as soon you are born, the first thing you get is your birth certificate. And it says American citizen. Even though you don't speak English.

## Discussion

The purpose of this work was to better understand the stressors faced by Puerto Rican men in the U.S. Northeast using a previously developed model used for minoritized populations. Our findings yielded expected parallels between our sample's stress processes and the pathways delineated by Meyer's MSM. Nevertheless, important deviations were present, particularly when considering movement and migration experiences and the historical context of U.S. colonialism.

Participating men referenced the complicated history of colonization by the U.S. in Puerto Rico as the genesis of collective misery for the Puerto Rican body politic. Our data indicates that these understandings were used by participants to contextualize their own current realities. This suggests that some participants use this legacy of colonialism and violence against Puerto Ricans—on the island and mainland—to make sense of their experience with everyday stressors, including those rooted in lived and vicarious prejudice, racism, discrimination, and marginalization. Importantly, the historical contexts played out in both stories of collective histories as Puerto Ricans, as well as in more individualized movement and migration stories. Importantly, participants were able to find parallels between their experiences and that of other colonized people by linking personal to political storied similarities. All told, the stories highlighted participants' own indigenous theorization that health-detrimental conditions in the present are part and parcel of a colonially violent past. In its current configuration, when the MSM is applied to the stress experience of the Puerto Rican population, particularly that of stateside Puerto Rican men, it falls short in etching out the often-obscured historical trajectories of stress, i.e., the health consequences of the continued colonial territory of Puerto Rico by the U.S. and the historical and present-day causes and ensuing stresses of resulting mass movement and migration. The MSM can aid in *describing* the structures and conditions that lead to stress, yet what it does not do is elucidate a historical lens to further probe an underlying issue: Why are so many Puerto Ricans living in the U.S. Northeast in the first place? Furthermore, why do they continue to emigrate from the island, which for many, this exodus has contributed to adverse health outcomes? The answer lies in a history laden with violence—the plunder and expropriation of people from their land by larger political economic forces (Spain, then the U.S.) and continued neoliberal policies and ensuing economic stagnancy, as well as climate injustices, that ravage livelihood on the island.

Others have adopted an historical lens to explain population-based health disparities: stress levels and adverse health outcomes among Black Americans cannot be understood without considering the history of enslavement (Dozier and Munn, 2020) and the afterlife of slavery (Davis, 2019); epidemics of communicable and non-communicable disease in South Africa cannot be understood without the history of colonial subjugation and apartheid (Coovadia et al., 2009), just as the history of Māori Indigenous people must be linked to the colonial history of New Zealand (Reid et al., 2019); disproportionate rates of diabetes in Indigenous populations cannot be understood without their shared experiences of colonization and expropriation of land (Fortier, 2008; Voaklander et al., 2020) and the brutal history of residential schools (Howard, 2014); and increased risk of cardiometabolic disease amongst Indigenous populations can also not be explained without considering the historical trauma of subjugation (Lewis et al., 2021). An expanded MSM model for Puerto Rican men adds to this literature and provides a theoretical underpinning and best-fit framework upon which to further strengthen and codify future intervention that gives weight to highlighting history and how this impacts the present. Probing deeper not only helps our collective approach to understand stress processes in marginalized Latinx communities in general (many of whom migrate to the U.S. due to imperial forces) but can potentially strengthen multi-level intervention approaches and our continued questioning of the larger and persistent political economic forces that drive inequities in health outcomes.

As Meyer argues, it is vital to have multilevel interventions. While we continue to push for individual coping mechanisms to address stress (such as therapy, healthy eating, and exercise) and community-based interventions (such as more resources to strengthen disadvantaged neighborhoods, build infrastructure, create jobs and affordable housing), we must also include the culpability of continued austerity measures and the U.S. colonial hold on Puerto Rico. Activism and advocacy amongst and alongside Puerto Rican communities must challenge continued colonial oppression. The U.S. treatment of the island is a critical determinant of stress-filled living conditions for Puerto Ricans on the island, subsequent stress-induced movement and migration, and consequent poor health outcomes for Puerto Ricans on the mainland. The U.S. has a responsibility to the Puerto Rican people, and thus interventions must not stop at individual or community levels.

A recent article in *Pedagogy in Health Promotion* argues that public health degree programs do little to teach about the historical origins of health inequities (Fleming, 2020). Yet, the historical connections to present-day health inequities are crucial for understanding the root causes of suffering and therein informing multilevel interventions (Dozier and Munn, 2020; Fleming, 2020). An historical perspective is essential to illuminate that “present-day inequities in the United States were constructed over time” (Fleming, 2020, p. 254), thereby strengthening both the analysis and approach to addressing disparities. A historical lens helps to bolster a wider structural analysis to health disparities and takes the emphasis off of individual blame and choice (Fleming, 2020). Rather, we place the individual within the context of their immediate environment and larger historical and structural factors of a given society to better understand the intricate pathways leading to the biological embodiment of stress.

Fleming's assertion calls to mind the work of medical anthropologist and physician Paul Farmer, who is well-known for his decades-long dedication to addressing global health inequities and examining the impact of historical structural violence on the creation and reproduction of preventable suffering and disease (Farmer, 1999, 2005). Suffering, he writes, “is ‘structured’ by historically given (and often economically driven) processes and forces that conspire—whether through routine, ritual, or, as is more commonly the case, the hard surfaces of life—to constrain agency” (1999, p. 40). Here, Farmer writes about how one cannot understand (or find concrete solutions to) the AIDS epidemic in Haiti without understanding the long history of colonization and political violence that has ravaged the country. Individually embodied suffering (whether that is physical and/or psychological) must be continually linked to both historical legacies and the consequent structural edifices of society.

### Strengths and Limitations

Historically speaking, Latino men are a difficult group to engage in health-related research (Rhodes et al., 2018; Valdez and Garcia, 2021). As a result, there is great strength stemming from the opportunity to engage Puerto Rican men in vulnerable and open dialogue about their lived experiences and the roots of their acute and chronic stressors. We believe that our approach to recruit and engage Latino men in community-based, non-clinical settings with a team of bilingual and bi-cultural peer mentors was key to our collective success. There is also a great deal of strength that stems from the rigor of our qualitative analysis and the findings they yield. Additionally, the insights shared by participants provide us with a paradigm shifting approach to examining stress processes in Latinx communities, which will be imperative to future intervention efforts with these populations.

Although the current study includes valuable insights of a seldom-engaged community to emphasize the importance of incorporating historical legacies within the MSM model, and thus, in future interventions to tackle health disparities, the study is not without its limitations. The work to expand the MSM to be responsive to the lived experiences of no/low-income Puerto Rican men was done prior to the emergence of COVID-19 and does not adequately account for the compounded effects of the pandemic on stressors and coping mechanisms. This is of particular importance, given that the Latino population in the U.S. has been disproportionately affected by the COVID-19 fallout (Shah et al., 2020; van Dorn et al., 2020). Another limitation, as with most qualitative inquiry, is the limited generalizability of our findings. The data collected in this study represent a limited geographical and cultural cross section of Latinx communities and, thus, cannot reflect the lived experiences of the vastly heterogeneous population of Latinxs living across the U.S. Nevertheless, as indicated in our data and supported by the literature, parallels in historical and current experiences with colonization and imperialism and resulting marginalization exist across Latinx communities, which warrants a closer confirmatory analysis. Future research should explore perceptions of the impact and influence of histories of subjugation and life trajectories of movement and migration on stress and health.

An additional consideration and potential limitation important to our understanding of the stress process lies at the intersection of historical context and the creation of Latinx manhood and masculinity. Research on the effects of self-conceptualizations and expressions of manhood and masculinity and its influence on stress continue to grow. Nevertheless, these notions were not explicitly present in the current data which warrant future inquiry that more precisely examines how a history of violent colonization, and the loss of power, agency, language, and culture also has an effect on the creation of masculinity which in turn has an effect on stress processing and coping.

## Conclusion

Our work adds to the literature a confirmation of how stressors in one specific minoritized community map onto a previously developed heuristic framework and allow us to use this framework with more confidence. Nevertheless, our ability to further contextualize the lived experiences and stressors of Puerto Rican men in the U.S., and the historical circumstances that shape their current realities, as well as their shared understandings of these realities is important to consider as we work to expand the MSM to guide intervention development. An expanded framework allows us to take stock of the past to understand our present and better map a just future. This is best exemplified by the words of Melendez (2003), one of the founders of the New York chapter of the Young Lords, as articulated in his memoir:

For all its influence on people's lives, history often seems more like a silent cloud than a guiding hand. No matter how subtle or silent, our history has a profound influence on our present and our future. If we search carefully enough, its path can be traced through the deeds and hopes of our ancestors to the very source and foundation of ourselves. You either claim your history or you lose authority over your future.

## Data Availability Statement

The raw data supporting the conclusions of this article will be made available by the authors, without undue reservation.

## Ethics Statement

The studies involving human participants were reviewed and approved by University of Massachusetts Amherst Institutional Review Board. The patients/participants provided their written informed consent to participate in this study.

## Author Contributions

LV conceptualized the study and conducted all data collection. LV, AM, and MB analyzed and interpreted the data. LV and AM wrote the initial draft of the manuscript. AG provided supervision and review and editing to the manuscript. All authors had full access to all the data in the study, take responsibility for the integrity and accuracy of the data analysis, contributed to read, and approved the final version of the manuscript.

## Funding

Research reported in this publication was supported by the National Institute on Minority Health and Health Disparities of the National Institutes of Health under Award Number R01MD010618.

## Author Disclaimer

The content is solely the responsibility of the authors and does not necessarily represent the official views of the National Institutes of Health.

## Conflict of Interest

The authors declare that the research was conducted in the absence of any commercial or financial relationships that could be construed as a potential conflict of interest.

## Publisher's Note

All claims expressed in this article are solely those of the authors and do not necessarily represent those of their affiliated organizations, or those of the publisher, the editors and the reviewers. Any product that may be evaluated in this article, or claim that may be made by its manufacturer, is not guaranteed or endorsed by the publisher.
